# Medical Society of the County of Erie

**Published:** 1896-03

**Authors:** Franklin C. Gram

**Affiliations:** Secretary


### Society Proceedings.

### MEDICAL SOCIETY OF THE COUNTY OF ERIE.

*Special Meeting, February 18, 1896.*

Reported by FRANKLIN C. GRAM, M. D., Secretary.

A SPECIAL meeting of the Medical Society of the County of Erie was held in the rooms of the Buffalo Academy of Medicine, February 18, 1896. Dr. J. G. THOMPSON, the president, called the meeting to order at 4 P. M., and Dr. METCALFE acted as secretary until the arrival of Dr. GRAM, a few minutes later.

Dr. M. D. MANN read a letter from the committee on legislation of the State Medical Society, condemning a bill now pending in the assembly and known as the Stanchfield bill, No. 728. This bill purports to favor higher medical education by extending the time of study to four years ; section 2 names several studies for examination, but prescribes no standard, thus leaving medical schools absolutely free to matriculate anyone who goes through the form of an examination ; section 3 exempts certain matriculates from an entrance examination ; section 4 makes provision in case of failure ; section 5 does away with all safeguards as to age, character and instruction ; section 6 postpones the restrictions of matriculation indefinitely if the candidate matriculates before 1897 ; and the final section may be construed to wipe out all the good and essential features of existing medical laws.

Remarks were made on the subject by Dr. Mann, Dr. H. R. Hopkins, Dr. Benedict, Dr. S. S. Greene, Dr. E. Storck and Dr. Lucien Howe. The opinion was expressed that while the four-years' term of study was a good feature, yet the remainder of the bill would more than counterbalance it ; and further, that this was, perhaps, the entering wedge for further legislation, which might wipe out all the good which has been accomplished after years of work in that direction.

On motion of Dr. HOPKINS, the following resolution was unanimously adopted :

WHEREAS, The Medical Society of the County of Erie, assembled in special meeting for the purpose, has heard the assembly bill No. 728, entitled "An Act to provide for four years' study of Medicine, etc." ; and

WHEREAS, This subject has been thoroughly discussed and considered ; and

WHEREAS, This society is in hearty sympathy with any proposition to extend the course of study of medicine to four years, but holds it to be of prime importance that the preliminary standards for medical students remain high and the enforcement in the hands of the state as now ; therefore be it

*Resolved*, That this society considers the above bill dangerous, retrogressive and inimical to the public health and the best interests of the medical profession, and that a committee be appointed to attend the hearing to oppose the same, and that we call upon our members of the legislature, senators and assemblymen, to use all honorable means to prevent its passage.

Dr. HOWE moved that a committee of three be appointed to attend the hearing at Albany, February 19th. This was carried and the Chair appointed Drs. Hopkins, Mann and Hubbell.

Dr. HOWE also called attention to the bill before the legislature proposing to legalise the practice of prescribing and fitting glasses by others than regular physicians in this state. A petition against this bill was signed by all the members present.

Health Commissioner WENDE asked all physicians who could, to attend the meeting of the ordinance committee of the board of aldermen and urge the adoption of an ordinance imposing the same conditions on others than qualified practitioners in regard to reporting contagious diseases and other illness.

The society adjourned at 5 P. M.

---

FORMULÆ FOR BLEPHARITIS.—Mittendorf recommends the following :

℞—Red oxide of mercury . . . . . . . . . . . . . . . . . grs. x.
Vaseline . . . . . . . . . . . . . . . . . . . . . . . . . . ℥ss.

M. Sig.—Apply to the edges of lids at bed-time.

Or, ℞—Ammoniated mercury . . . . . . . . . . . . . . . . . grs. xx.
Powdered camphor . . . . . . . . . . . . . . . . . . grs. x.
Vaseline . . . . . . . . . . . . . . . . . . . . . . . . . fl. ℥ss.

M. Sig.—Apply at night.

Or, ℞—Solution of subacetate of lead . . . . . . . . . . gtts. x.
Ointment of rose water . . . . . . . . . . . . . . . ʒiij.

M. Sig.—To be used for more chronic forms of marginal blepharitis.

